# Relationship between serum cortisol level and degree of false lumen thrombosis in patients with uncomplicated type B aortic dissection

**DOI:** 10.1038/s41598-018-19691-6

**Published:** 2018-01-19

**Authors:** Yani Wu, Yudong Sun, Xiaolong Wei, Lei Zhang, Tonglei Han, Zhiqing Zhao, Jian Zhou, Zaiping Jing

**Affiliations:** 1 Department of Vascular Surgery, Changhai Hospital, Second Military Medical University, Shanghai, 200433 China; 2 Present Address: Department of Breast and Thyroid Surgery, Changhai Hospital, Second Military Medical University, Shanghai, China

## Abstract

Partial thrombosis of the false lumen in uncomplicated type B aortic dissection (uTBAD) is associated with an increased late mortality risk. Whether the serum cortisol level can affect false lumen thrombosis in patients with uTBAD has not been well characterized. This study was performed on 87 patients with uTBAD. A curve-fitting method was used to analyze the relationship between serum cortisol and partial thrombosis of false lumen. Univariate and multivariate logistic regression analyses were used to identify false lumen partial thrombosis–associated serum cortisol. Curve-fitting’s result revealed a characteristic U shape, and 14.0 µg/dL was considered as the cutoff point for serum cortisol. The results of univariate and multivariate logistic regression analyses suggested that when trisecting the serum cortisol level into three parts, the low and high levels of serum cortisol could significantly affect the occurrence of false lumen partial thrombosis compared with the middle level. The odds ratio value of the low and high levels of serum cortisol was 6.12 and 4.65, respectively, in the univariate analysis, and 24.32 and 3.93, respectively, in the multivariate analysis. Low or high levels of serum cortisol might influence the natural result of uTBAD through affecting the false lumen thrombosis.

## Introduction

Aortic dissection (AD) remains a catastrophic cardiovascular disease with increasing incidence, high mortality, and severe complications^1,2^. Patients with uncomplicated type B aortic dissection (uTBAD) without aorta rupture or organ ischemia are usually treated medically, with 90% surviving on effective antihypertensive therapy^3^. The critically important clinical measurement of uTBAD is false lumen thrombosis. Thrombus formation is a complex biological process involving many chemical and biological species, transport phenomena, and kinetic processes. Some studies have reported a strong relationship of the thrombus state of false lumen with the late events of AD^4–6^. Moreover, patients with type B aortic dissection (TBAD) with partial false lumen thrombosis have been found to have an increased late mortality risk^7^. However, most of the studies focused on the relationship between fluid dynamics and false lumen thrombus in AD and neglected the complex network of biochemical mechanisms involved in this process.

The hypothalamic–pituitary–adrenal (HPA) axis is involved in the stress reaction by releasing cortisol. Disturbances in the HPA axis function in the case of cortisol excess or deficiency may adversely affect the cardiovascular system both indirectly by inducing hypertension, insulin resistance, or dyslipidemia, and directly by interactions with cellular pathways important in the development of atherosclerosis and thrombosis. Recent observations indicated that dysregulated cortisol concentration was linked with physical function^8^ and level of consciousness^9^. A number of studies have reported that cortisol triggers cardiovascular events such as stroke^10–12^, coronary artery disease^13,14^, and myocardial infarction^15–17^. However, it is still unclear whether serum cortisol affects the prognosis of patients with AD.

Cortisol is thought to have both direct and indirect effects on the vasculature system, leading to metabolic disorders and hence contributing to the high risk of arterial and venous thrombosis^18–20^. A population-based case–control study in Denmark demonstrated that the risk of venous thromboembolism was enhanced among exogenous glucocorticoid users^21^. It is well known that thrombin generation is counterbalanced by stoichiometric inhibitors such as tissue factor pathway inhibitor (TFPI), which is the principal regulator of the initiation phase^22^. Studies have reported a decrease in the levels of TFPI and prothrombotic state in hypercortisolic patients^23^. Cortisol impairs fibrinolytic capacity, upregulating the synthesis of plasminogen activator inhibitor type 1. These changes result in an impaired thrombin generation that has a prothrombotic effect^24^.

The aim of this study was to evaluate the relationship between serum cortisol level and degree of false lumen thrombosis in patients with uTBAD without the thoracic endovascular aortic repair. Furthermore, it analyzed the serum cortisol level as a potential prognostic factor affecting the natural result of uTBAD.

## Methods

### Study Population

**Figure 1:**
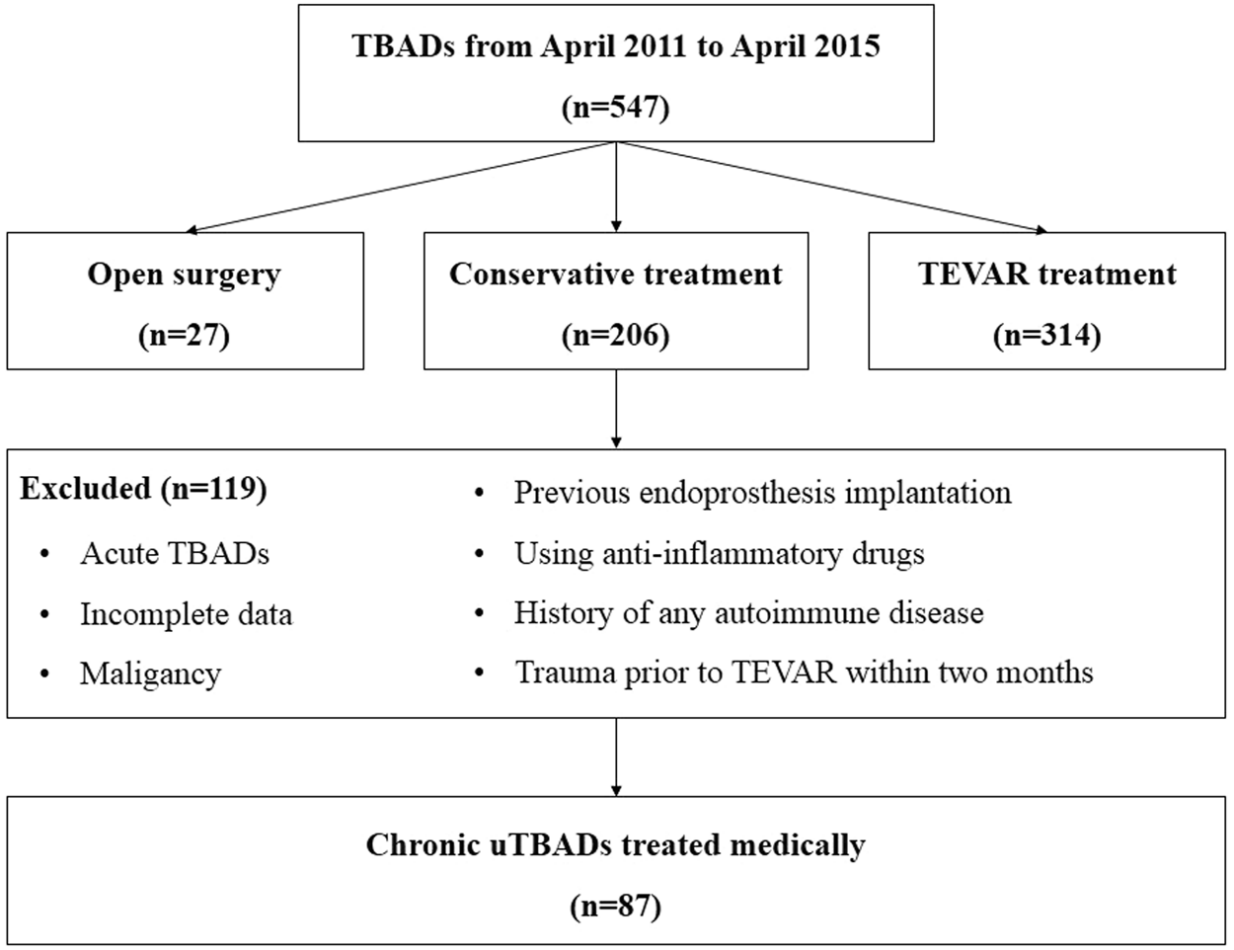
Flow diagram for study identification and inclusion.

The study protocol complied with the declaration of Helsinki and was approved by the ethics committee of Changhai Hospital. All patients provided their written informed consent forms before participating in this study. From April 2011 to April 2015, a total of 547 TBAD patients were admitted to Shanghai Changhai hospital for their first-ever TBAD. The diagnosis of TBAD was defined according to computer tomography angiography (CTA) on a 64-slice CT scan (Siemens, Munich, Germany) within 24 hours after admission. Patients with surgical treatment including open and TEVAR, acute TBAD and other causes of activation of the HPA axis (a history of acute or chronic infections or surgical procedures within the last three weeks), Marfan syndrome, connective tissue disorder, malignancy, febrile disorders, acute or chronic inflammatory diseases were excluded from this study. We also excluded patients receiving all types of steroids, immunosuppressive agents and psychotropic drugs. (Figure 1).

### Medical therapy

Careful control of blood pressure is thought to be classical standards for treatment of uTBAD. All patients in our study were treated medically at the time of diagnosis confirmation. The medication was achieved by oral antihypertensive and it was administered either alone or in combination to maintain systolic blood pressure below 140 mmHg. Beta-blockers were administered rountinely unless contraindicated, although the selection was left to the discretion of each clinician.

### Hormone analysis and laboratory measurements

Blood samples were collected to determinate the biochemical parameters and cortisol at 8:00 AM on the next day morning after admission. The blood samples were centrifuged and serum was stored at −80 °C. Serum cortisol was measured by immunoassay (Unicel DxI 800, BECKMAN COULTER, USA). The normal range for morning serum cortisol concentration in our hospital laboratory is 8.7 to 22.4 ug/dl. Blood was centrifuged to obtain plasma and plasma aliquots for ghrelin assay were acidified with HCl to 0.05 N to prevent ghrelin degradation. Hyperion MR III double antibody sandwich ELISA (San Antonio, Texas, USA) was used to measure ghrelin according to the manufacturer’s instructions. ACTH levels were measured by enzyme-linked immuno sorbent assay (ELISA) kits (Phoenix Pharmaceuticals, Burlingame, CA). Routine laboratory tests (serum glucose, total cholesterol, triglycerides, HDL cholesterol, VDL cholesterol, CRP, ESR, D-dimer, leucocyte counts, ALT, AST, LDH, FDP, Prothrombin time, Thrombin time) were also performed in all participants at admission. The measurement of these indexes were repeated at one, three and six month during follow-up. The blood samples were collected at 8:00 AM on the day morning they come to the out-patient clinic. We use the mean value of the four-time measurement to do further analysis.

The following information were recorded: age, gender, Body mass index (BMI), blood pressure on admission, history of conventional vascular risk factors (alcohol abuse, smoking habit, hypertension) and medical history (diabetes mellitus, coronary artery disease, peripheral vascular disease, Hypohepatia, Renal failure, Interventional surgery).

### Computed Tomography Image Analysis

CTA examination were arranged in patients at 6 month follow-up point. Parameters were obtained with the help of dedicated three-dimensional workstation (Aquarius WS 3.7.0.13, TeraRecon Inc, San Mateo, Calif). Briefly, enhanced aortic lumen was reconstructed by volume rendering technique. Then the aortic segment where thrombosis of the false lumen exists was extracted by cropping area of interest. Volume measurements were automatically done using the “volume measure” function. The new-onset thrombosis volume in false lumen was defined as the reduced volume in false lumen between the first admission and the six months follow-up aortic volume at the same segment. Data were measured by an experienced vascular radiologist at least three times on the initial preoperative CTA images. The status of the false lumen was classified as complete thrombosis if no flow was present, as partial thrombosis if both flow and thrombus were present and as patent if flow was present in the absence of thrombus. Thus, according to the status of the false lumen, the patients were divided into two groups. The following parameters were also measured or counted at the same time: the maximum diameters of abdominal aorta and abdominal FL, the numbers of visceral arteries from the FL, the numbers of intimal tears.

### Statistical Analysis

All analyses were performed using Empower(R) (www.empowerstats.com, X&Y solutions, inc. Boston MA) and R (http://www.R-project.org). The categorical variables are presented as numbers (percentages) and all the continuous variables as means ± standard error or interquartile range, depending on variable distribution. Categorical variables were compared using chi-squared or Fisher’s exact test, and Student’s t test or Wilcoxon rank-sum test for numeric variables. The association between serum cortisol and the thrombosis degree of false lumen was examined using the smoothing plot. Univariate analyses were used to evaluate the impact of laboratory examination index on the occurrence of partial false lumen thrombosis for each patient. The p-value of P < 0.05 was considered significant.

## Results

### The characteristics of the participants

In total, 87 patients with uTBAD were enrolled in our study. Patients were divided into two groups based on their false lumen thrombosis status at six month follow-up. There were 43 (49.4%) patients in the group of complete thrombosis or patent false lumen and 44 (50.6%) patients in the group of partial thrombosis. The differences of the basic characteristics among the two groups are listed in Table 1. Serum cortisol did not significantly differ between the two groups. The visceral arteries from false lumen was significantly higher in partial thrombosis group. There were no statistical differences in the other demographic and clinical data among the two groups.

**Table 1:** Basic Characteristics of all patients according to their false lumen status.

Variable	None partial thrombosis (n = 43)	Partial thrombosis (n = 44)	P value
Age, yrs	58.56 ± 12.61	61.30 ± 12.63	0.315
Male, n(%)	36 (83.72%)	37 (84.09%)	0.963
Body mass index, kg/m^2^	22.73 ± 3.81	23.29 ± 3.14	0.186
Smoking, n(%)	14 (32.56%)	12 (27.27%)	0.590
Hypertension, (n%)	31 (72.09%)	30 (68.18%)	0.690
CAD, n(%)	7 (16.28%)	4 (9.09%)	0.313
Diabetes mellitus, n(%)	7 (16.28%)	12 (27.27%)	0.215
PVD, n(%)	5 (11.63%)	5 (11.36%)	0.969
Hypohepatia, n(%)	3 (6.98%)	8 (18.18%)	0.116
Renal failure, n(%)	3 (6.98%)	4 (9.09%)	0.717
Interventional surgery, n(%)	3 (6.98%)	2 (4.55%)	0.626
ACTH, pg/ml	25.68 ± 14.19	29.14 ± 22.12	0.389
Serum Cortisol, ug/dl	13.27 ± 5.50	14.10 ± 8.72	0.598
Serum glucose, mmol/L	6.03 ± 1.51	6.60 ± 1.63	0.091
Total cholesterol, mmol/L	4.29 ± 0.69	4.26 ± 0.55	0.827
Triglyceride, mmol/L	1.50 ± 0.50	1.43 ± 0.62	0.548
CRP, mg/L	46.04 ± 34.69	42.54 ± 28.29	0.607
ESR, mm/h	14.81 ± 10.78	16.32 ± 11.29	0.527
D-dimer, mg/L	3.72 ± 2.52	6.24 ± 6.97	0.029
Interleukin-6, pg/ml	16.91 ± 19.80	16.69 ± 15.68	0.954
FDP, ug/ml	13.14 ± 10.51	15.92 ± 18.99	0.402
ALT, U/L	27.28 ± 20.01	24.86 ± 13.61	0.511
AST, U/L	26.26 ± 13.66	25.02 ± 7.66	0.604
Prothrombin time, s	12.84 ± 0.73	13.03 ± 0.82	0.271
PreMax on Ab (mm)			
Aorta	38.23 ± 6.20	38.63 ± 10.01	0.820
FL	24.06 ± 5.70	27.16 ± 9.91	0.078
Intimal tear	2.26 ± 1.24	2.75 ± 1.28	0.070
Visceral branches from FL	1.53 ± 1.22	2.41 ± 0.97	<0.001

### Serum cortisol and partial thrombosis of false lumen

**Figure 2:**
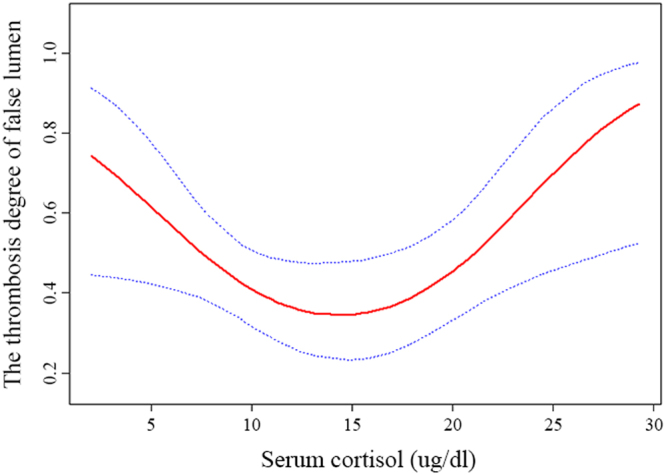
The relationship between serum cortisol and the false lumen partial thrombosis*. *Adjusted for age, gender, body mass index, smoking, hypertension, intimal tears, visceral arteries from the false lumen.

Curve fitting method was used to clarify the relationship between serum cortisol and the partial thrombosis of false lumen when adjusted for age, gender, body mass index, smoking, hypertension, intimal tears, visceral arteries from the FL and its characteristic was presented in Fig. 2. It represents a U-shape characteristic and the false lumen with low or high level of serum cortisol presents partial thrombosis. We regarded “0” as none or complete thrombosis of FL, “1” as partial thrombosis of FL. The risk of false lumen partial thrombosis decreased with the serum cortisol level up to the turning point (14.0 ug/dl). (OR 0.86, 95%CI 0.6–0.96, p = 0.02). When the serum cortisol level was ≥14.0, the risk of false lumen partial thrombosis increased. (OR 1.24, 95%CI 1.05–1.46, p = 0.01) (Table 2).

**Table 2:** Threshold effect analysis of serum cortisol on partial thrombosis of false lumen using piecewise linear regression.

Inflection point of serum cortisol	Odds ratio^a^ (95% CI)	P value
<14.0	0.86 (0.66, 0.96)	0.02
≥14.0	1.24 (1.05, 1.46)	0.01

A 14-U threshold for the serum cortisol existed for risk of false lumen partial thrombosis.

^a^Adjusted: Age, Gender, Body mass index, Smoking, Hypertension, Intimal tear, Visceral branches from false lumen.

### Systemic findings associated with partial thrombosis in the false lumen

Recent studies showed that TBAD patients with partial false lumen thrombosis had an increased late mortality risk^7^. In order to explore the risk factor of partial thrombosis of false lumen, we did univariate and multivariate logistic regression analysis and the result was showed in Table 3 when adjusted for age, gender, body mass index, smoking, hypertension, intimal tears, visceral arteries from the FL. We trisect the serum cortisol level into three parts (low, middle and high) and a total of 10.97 and 15.80 were considered as the cut point for serum cortisol. We use middle level of serum cortisol as control. It revealed that low and high level of serum cortisol can significantly affect the occurrence of false lumen partial thrombosis when compared with the middle level. The odds ratio values of the low and high level of serum cortisol are 6.12 and 4.65 respectively in univariate analysis, 24.32 and 3.93 respectively in multivariate analysis.

**Table 3:** Risk factors for partial thrombosis in the false lumen*. *Adjusted for Age; Gender; Body mass index; Smoking; Hypertension; Intimal tear; Visceral branches from false lumen.

Variable	Univariate analysis			Multivariate analysis		
	OR	95% CI	P value	OR	95% CI	P value
FDP, ug/ml	1.00	0.97–1.04	0.80	1.00	0.98–1.02	0.92
Serum Cortisol, ug/dl						
middle	1.0	1.0	1.0	1.0	1.0	1.0
low	6.12	1.29–28.97	0.02	24.32	2.20–268.36	<0.01
high	4.65	1.05–20.53	0.04	3.93	0.62–24.78	0.04
ACTH	1.01	0.98–1.04	0.52	1.05	1.00–1.10	0.07
ESR	1.00	0.95–1.05	0.96	0.97	0.90–1.03	0.29
CRP	1.00	0.98–1.01	0.64	1.00	0.98–1.02	0.92
Interleukin 6	1.02	0.99–1.05	0.26	1.01	0.97–1.05	0.73
D-dimer	1.13	0.99–1.28	0.07	1.22	0.86–1.73	0.26
Total cholesterol	0.58	0.25–1.38	0.22	0.23	0.05–0.94	0.09
Serum glucose	1.46	1.00–2.12	0.05	1.57	0.92–2.68	0.10

FDP, fibrin degradation product; ACTH, adrenocorticotropic hormone; ESR, erythrocyte sedimentation rate; CRP, C- reactive protein.

## Discussion

The present set of findings supported a link between serum cortisol and degree of false lumen thrombosis in patients with uncomplicated TBAD. The results showed that patients with uTBAD having low or high level of serum cortisol presented more probability of the occurrence of false lumen partial thrombosis compared with the middle level. These results implicated the contributory role of cortisol in the thrombosis of false lumen during the development of uTBAD.

Serum cortisol has a close relationship with human health by releasing cortisol. Studies have found that the disorder of serum cortisol might affect the cardiovascular pathological process through regulating blood pressure, lipid metabolism, and insulin resistance^25,26^. A study explored the relationship between women’s morning serum cortisol and coronary artery disease and revealed that increased cortisol levels might contribute to the occurrence of atherosclerosis^27^. In another small study of 105 subjects, the cortisol levels in blood in the morning prior to coronary angiography, circumstances described by the authors as “high anticipatory stress,” were positively correlated with the severity of coronary artery disease, independent of other cardiovascular risk factors^13^. However, Reynolds *et al*. measured the plasma cortisol level in 278 subjects with suspected coronary artery disease (CAD) in the morning under unstressed conditions^28^. They found that the circulating cortisol levels tended to be lower in those with confirmed vessel disease on angiography and in those requiring intervention following angiography. These findings suggested that the responsiveness of the HPA axis might be more predictive of CAD compared with the basal activity of HPA axis. Interestingly, Hatzaras *et al*. retrospectively analyzed the inciting events in 90 patients with acute aortic dissection and found that severe physical and emotional stressful events could be identified as precipitating factors in the acute onset of AD in more than two thirds of patients^29^. However, whether serum cortisol levels predict the process of TBAD is unclear. The thrombosis status of the false lumen in TBAD was reported to be the strongest independent predictor of outcomes^30^. Therefore, the present study focused on the relationship between serum cortisol and partial thrombosis of false lumen in patients with uTBAD.

Blood coagulation, changes in the fibrinolytic system, and the morphologic status of TBAD are involved in the development of false lumen thrombosis. The potential factors, including fibrin degradation product level, D-dimer level, maximal diameter of aorta and false lumen, number of intimal tears, and visceral branches from the false lumen, might affect false lumen remodeling^31–33^. Further, hypercortisolism could stimulate thrombus formation by increasing the levels of coagulation factors and decreasing fibrinolysis; however, the results have been inconsistent^34–36^. Conversely, glucocorticoids have been supposed to suppress platelet activation by downregulating the synthesis of endothelial prostacyclin^37,38^. Therefore, cortisol might exert both promoting and inhibitory effects on thrombus formation. In fact, cortisol is an HPA axis–related hormone with a strong circadian rhythm where the levels typically peak in the morning hours and decline across the day. Meanwhile, the Fukuoka *et al*. suggested that autonomous cortisol secretion, rather than daily total secretion, might be associated with thrombus formation^39^. It is possible that these inverse effects occur at different cortisol levels, with beneficial effects occurring mainly within the physiological range and harm imposed by only prolonged conditions of cortisol excess.

In addition, physiological stress could induce metabolic changes such as hypertension by regulating the HPA axis. Moreover, recent studies confirmed that high blood pressure variability might be associated with initial thrombosis of false lumen in TBAD^40^. Thus, it is believed that physiological stress, namely serum cortisol, may affect false lumen thrombosis in patients with TBAD.

### Limitations

The present study clearly demonstrated the correlations between serum cortisol level and occurrence of false lumen thrombosis in patients with uTBAD. However, the exact mechanisms involved in the formation of false lumen thrombosis by serum cortisol remain unclear. The principal limitations of the present study were the small number of subjects available in a single center and the lack of long-term follow-up. Also, the study simply tested the serum cortisol level in the morning, although the fluctuation in cortisol was the highest in about 30 min after waking up in the morning and the lowest at midnight. Furthermore, this was only a preliminary study. Hence, these results need to be replicated in more prospective studies to ensure whether they are stable and valid.

## Conclusions

In summary, this novel study revealed the relationship between serum cortisol level and partial thrombosis of false lumen in patients with uTBAD. Low or high levels of serum cortisol might influence the natural result of uTBAD through affecting false lumen thrombosis. Maintaining serum cortisol to a middle level may be beneficial by inhibiting the occurrence of false lumen partial thrombosis. Further studies on this subject might provide new biological markers to assess the development of false lumen thrombosis in patients with uTBAD.
